# Synthesis of a Peptide Derivative of MicrocinJ25 and Evaluation of Antibacterial and Biological Activities

**DOI:** 10.22037/ijpr.2019.1100750

**Published:** 2019

**Authors:** Maryam Mazaheri Tehrani, Mostafa Erfani, Nour Amirmozafari, Taher Nejadsattari

**Affiliations:** a *Department of Microbiology, Science and Research Branch, Islamic Azad University, Tehran, Iran.*; b *Radiation Application Research School, Nuclear Science and Technology Research Institute (NSTRI), Tehran, Iran.*; c *Department of Microbiology, School of Medicine, Iran University of Medical Sciences, Tehran, Iran. *; d *Department of Biology, Science and Research Branch, Islamic Azad University, Tehran, Iran.*

**Keywords:** MicrocinJ25 derivative, Synthesis, Biological activity, E. coli, Cytotoxicity

## Abstract

MicrocinJ25 (MccJ25) is a small ribosomally synthesized antimicrobial peptide that is produced by *Enterobacteriacea* family especially *E. coli*. The present study focuses on preparation and evaluation of *in-vitro* antimicrobial and biological properties of a new peptide derived from MccJ25. We prepared a MccJ25-derived peptide containing 14 amino acids and a single intra-molecular disulfide bond according to solid phase synthesis strategy. The purified peptide was characterized by Liquid chromatography-mass spectrometry (LC-MS) and Fourier Transform Infrared (FTIR) spectroscopy. 96-well microdilution plate assay was exerted for determination of minimum inhibitory concentration (MIC) of peptide against different bacterial strains. Cytotoxicity of the peptide derivative on HT-29 cell line assayed using MTT test. The final peptide successfully was prepared with purity more than 99.8% as determined by analytical HPLC. The evaluation of antibacterial activity of the peptide against Gram-positive and Gram- negative bacteria revealed that the peptide was very effective against *E. coli* 35218 with minimum inhibitory concentration (MIC) at dose 3.9 µM. The hemolytic activity toward human erythrocytes was very minimal below 0.3%. The cell viability percentage of HT-29 cell line after 24 h of contact with the peptide was more than 83%. The high sensitivity of *E. coli* strain to this new peptide derived from MccJ25 and through minimal toxicity to cancerous cell, suggesting that above synthesized peptide could be considered as a bioactive compound for further investigations.

## Introduction

Antimicrobial peptides (AMPs) are one of the diverse groups of host innate defense peptides that are produced by virtually all forms of life: animals, plants, and microorganisms (1-6). Those peptides produced by bacterial strains including bacteriocins from Gram-positive bacteria and colicins/microcins from Gram-negative bacteria are low molecular weights of antimicrobial peptides that are ribosomally secreted during the primary phase of bacterial growth (5-8). AMPs from bacteria are mainly active against closely phylogenetically related species of the producing strains with different action modes of killing, whereas cause little or no damage to the producer bacterium due to the existence of intra-immunity systems and/or post-transcriptional modifications (1, 2, 9). 

Microcins are oligopeptides below 10 KDa in weight, produced mostly by members of *Enterobacteriacea* family. MicrocinJ25 (MccJ25), a highly hydrophobic cyclic peptide < 5 kDa in weight (2106 Da), belongs to the class I of microcins that have extensive post-translation modification in their precursor peptide prior to become a mature active form of microcin. During the last decade, the structure and mode of action of MccJ25 against Gram-negative bacteria has been studied in depth and known well (10-15). 

MccJ25 consists of 21 amino acid residues with a unique compact threaded topology (lasso folding) which is produced by some strains of *Escherichia coli* that harboring a plasmid–borne synthesis, maturation and export systems. Four plasmid genes containing mcjA, mcjB, mcjC, and mcjD are responsible for synthesis, export, and immunity of MccJ25 peptide. The lasso structure is a consequence of mcjB and mcjC genes function on mcjA gene product forming the term lariat-protoknot ring (lactam linkage between the N-terminal Gly^1^ and the γ-carboxyl of the side chain of Glu^8^ creating an eight residue - ring and passing the C-terminal tail through the ring) (13-15). Its high stability at high temperatures and resistance to proteolysis conditions are due to the lasso structure (16-19). MccJ25 disrupts cytoplasmic membrane respiratory chain and furthermore targets cellular DNA-dependent RNA polymerase (RNAP) by binding to the secondary channel of enzyme to block formation of transcription complex in sensitive *E. coli* strains (10, 12, 14-19). The antibiotic activity of MccJ25 is mainly directed to the *Enterobacteriaceae *family, including several pathogenic *Escherichia coli*, *Salmonella, *and *Shigella* strains (11, 14) The recent results of structure-activity analysis studies of lasso peptide MccJ25 suggested that the substitutions of amino acid residues at most of the positions of 21-amino acid sequence in natural form of MccJ25 can be tolerated (13, 18, 20). The structure of MccJ25 has two distinct regions including the ring and β-hairpin loop formed by the tail. Each of the structural regions has a particular role in the mechanism of the peptide’s antimicrobial activity. The β-hairpin loop is involved in recognition and import of MccJ25 by the outer and inner membrane proteins FhuA (iron receptor) and SbmA respectively into sensitive bacterial strains (21-24). The ring region of MccJ25 is critical for RNAP inhibition (8-24). Because of having high stability at extreme conditions, MccJ25 can be a valuable target for designing different synthetic analogues of the native peptide for application in clinical medicine. Up to now, ongoing researches on MccJ25-analogues could have provided more effective antimicrobial peptide drugs or interesting information on MccJ25 structure. The recent studies by Soudy *et al*. (25) and Hemmami *et al*. (26) have shown that it is possible to chemically synthesize MccJ25-peptide derivatives, lacking lasso folding, with different antibacterial properties. However, based on our knowledge, there are few reports in the literature on the synthesis of MccJ25 analogues with different antibacterial and biological activities for clinical application as an antimicrobial agent (27-29). 

The aim of the current study was the preparation and purification of a new cyclic peptide derivative of antimicrobial MccJ25 containing a disulfide bond as follow: [cyclo (Cys^1^-Gly^2^-Ala^3^-Gly^4^-His^5^-Val^6^-Pro^7^-Cys^8^)-Tyr^9^-D-Tyr^10^-GABA^11^-D-Phe^12^-Tyr^13^-Gly^14^]. The potency of the synthesized peptide against some bacterial strains from microbial collections was evaluated. Furthermore, the biological activity and cytotoxicity of the peptide as a compound derived from a natural antibiotic with bacterial origin was investigated.

## Experimental

All chemicals used in this study were of analytical grade purity and used as received. 2-chlorotrityl chloride resin (100-200 mesh) and N-α-fluorenylmethyloxy carbonyl (Fmoc) amino acid derivatives were purchased from NovaBiochem (Laufelfinegen, Switzerland). The necessary solvents with peptide grade synthesis were purchased from Sigma-Aldrich Company (St. Louis, MO, USA). The microbial culture media, trypticase soy agar (TSA), trypticase soy broth (TSB) and heart infusion broth (BHI) were commercially available from Merck (Germany). The cell culture medium RPMI 1640 supplemented with 10% fetal bovine serum (FBS), amino acids, vitamins and antibiotics from Gibco (Germany) was used for culturing cell line HT-29.

*Preparation of microcinJ25-peptide derivative*

The protected peptide was constructed using standard Fmoc-based solid phase synthesis on 2-chlorotrityl [(2-Cl) Trt] resin with substitution, 1.4 mmol/g (30). The amino acid derivatives with and without side chain protecting groups were respectively as: Fmoc-Cys(Acm)-OH, Fmoc-Gly-OH, Fmoc-Ala-OH, Fmoc-Gly-OH, Fmoc-His(trityl)-OH, Fmoc-Val-OH, Fmoc-Pro-OH, Fmoc-Cys(Acm)-OH, Fmoc-Tyr(tBu)-OH, Fmoc-D-Tyr(tBu)-OH, Fmoc-GABA-OH, Fmoc-D-Phe-OH, Fmoc-Tyr-(tBu)-OH, Fmoc- Gly-OH. Briefly, peptide bond formation between amino acids was made in the presence of 3 mole excess Fmoc-amino acid, 3 mole excess N-hydroxybenzotriazole (HOBt), 3 mole excess diisopropylcarbodiimide (DIC) and 9 mole excess diisopropylethylamine (DIPEA) in dimethylformamide (DMF). Completeness of amino acids coupling was monitored by the Kaiser test. The Fmoc protecting groups were removed by treatment with 20% piperidine solution (v/v) in DMF (10 mL). The synthesized peptide was cleaved from resin by the cleavage solution containing 20% trifluoroethanol (TFE), 0.5% trifluoroacetic acid (TFA), and 0.5% water (H2O) in dichloromethane (20 mL), subsequently incubated at room temperature for 30 min. Thereafter formation of disulfide bond between first and eighth cysteine amino acids was performed by iodine oxidation method. After dissolving Cys-Acm peptide (26 mmol) in 10% aqueous methanol (80 mL), the iodine solution (380 mg I_2_ in 2 mL Methanol) was added. After 30 min vigorous stirring, quench of iodine was performed by addition of 0.5 M ascorbic acid (0.44 g, 5 mL). The mixture was evaporated under reduced pressure to dryness and redissolved in 40 mL DCM and 10 mL H_2_O. Cyclic peptide was extracted in DCM phase and dried under reduced pressure. The side chain deprotection was performed by dissolving the peptide in a mixture of TFA (92.5%) /Thioanisole (2.5%)/ Triisopropylsilane (2.5%)/ H_2_O (2.5%) for 30 min at 25 ˚C. After removing the organic solvents in vacuum, the crude product was precipitated in cold petroleum ether and Diisopropyl Ether (50:50). 

Purification of the peptide performed by a semi-preparative HPLC system (Sykam S7131, Eresing, Germany) using column VP Nucleosil 100-5 C_18_ (250×21 mm), flow: 15 mL/min with a variable wavelength UV detector operating at λ=214 nm. The mobile phase consisted of 0.1% trifluroacetic acid (TFA)/H_2_O (solvent A) and acetonitrile (solvent B) with gradient: 0 to 5 min, 70% A; 5 to 15 min, 70% to 30% A; 15 to 20 min, 30% A. Finally, the purified peptide was characterized by analytical HPLC system using a C18 column (CC 250/4.6 Reprosil-pur ODS-3.5 µm), flow: 1 mL/min; λ=280 nm (0.1 % trifluoroacetic acid/water (Solvent A) and acetonitrile (Solvent B) and the gradient: 0 to 5 min, 95% A, 5 to 25 min, 95% to 0% A, 25 to 27 min, 0% A, 27 to 30 min 0% to 95% A). The peptide was stored at -20 ˚C until use.

*Structural determination of MccJ25 peptide derivative*

 For identification of peptide, LC-MS was performed by using Agilent G6410 triple Quadra pole mass spectrometer instrument in positive electrospray ionization (ESI) mode with Nebulizer: 15psi and LC condition as follows: Peptide solution (5 µL) was injected to LC- mass spectrometer using C_18 _column and mobile phase consisted of water/0.1%formic acid (solvent A) and methanol (solvent B). The gradient used a flow rate of 0.3 ml/min with a column temperature of 25 ˚C in A: B ratio of: 50:50. For further confirmation, FTIR spectrum of the product was recorded by a Brucker Vector 22 spectrometer instrument (Germany). 

*Antibacterial activity assay*

*Bacterial strains and growth conditions*

All bacteria were obtained from a microbial laboratory collection (Iran University of Medical Sciences). All strains were maintained in 20% glycerol/Brain Heart Infusion broth (Merck, Germany) at -70 ˚C. Two Gram-positive bacteria (*Staphylococcus aureus* ATCC25923, *Staphylococcus aureus* PTCC1112) and four Gram- negative bacteria (*Salmonella enterica* PTCC1709, *Salmonella typhimurium* ATCC14028, *Escherichia coli* ATCC35218 and *Escherichia coli *ATCC8739) were used in this study. All selected bacterial strains were grown aerobically in Trypticase Soy Broth (TSB) (Merck, Germany) at 35-37 ˚C. Stock cultures of the bacterial strains were maintained on Trypticase Soy Agar (TSA) (Merck, Germany) slants and stored at 4 ˚C. The strains were sub-cultured in fresh media at every experiment interval.

*Bacterial suspension standardization*

A McFarland 0.5 standard was prepared by mixing 99.5 mL of 0.18 M sulfuric acid (1% v/v) with 0.5 mL of 0.048 M (1.175%) barium chloride and was dispensed into screw cap glass tubes. The tubes were tightly sealed and stored under dark conditions at room temperature. The standard tubes were agitated on a vortex mixer before use. The McFarland 0.5 standard provides turbidity (OD_630_ = 0.08-0.1) comparable to that of a bacterial suspension containing approximately 1-2× 10^8^ colony-forming units (CFU/mL) (31).

*Preparation of bacterial suspension*

For preparation of bacterial suspension, 4-5 colonies of overnight culture of the test bacterial strain were transferred to 5 mL of a liquid growth medium (TSB) and the tubes (in a similar sized glass tube to the standard) were incubated at 35-37 ˚C for 2-3 h. After incubation, the turbidity of suspension was adjusted to 0.5 McFarland tubes by using UV/visible spectrophotometer (Biowave П, Biochrom,China).

*Determination of Minimal Inhibitory Concentration (MIC)*

The broth micro-dilution method using sterile 96-well microplate was performed for evaluation of minimal inhibitory concentration (MIC) of the peptide derivative as recommended by Clinical and Laboratory Standards Institute (CLSI, formerly NCCLS) (31). A stock solution of peptide (422 µM) was prepared in ultra-pure deionized water and then the different concentrations of the peptide on the basis of serial two fold dilutions ranging from 0.97-250 µM were obtained from stock solution. The microplate wells were loaded with 100 µL of bacterial suspension (1.5-2× 10^8 ^CFU/mL in TSB medium, OD_630_= 0.09-0.1) and followed by the addition of 10 µl peptide solution. Each peptide concentration was tested in triplicate on microplate. Wells containing bacterial cells in medium without peptide (positive control) and wells containing TSB medium and peptide without bacterial cells (negative control) were included on the same plate. The blank wells were loaded with TSB medium. The optical density of the plate was measured at 630 nm by using a microplate reader (BioTek, USA) at two steps as follows: after loading the wells and after 24 h incubation at 37 ˚C, respectively. MIC values were determined as the lowest peptide concentration that caused 100% inhibition of bacterial growth based on optical density of each well. Commercial antibiotic gentamicin at concentration (0.97-500 µM) was used as a standard for evaluation of MIC. In addition, the inhibitory effect of the highest concentration of peptide (250 µM) on bacterial growth (bacterial suspension containing 1.5-2× 10^8^ CFU/mL) was evaluated for 24 h incubation at 35-37 ˚C. 

*Cell culture*

HT-29 tumor cell line used in this study was purchased from Pasture Institute of Iran. The cells were maintained in RPMI 1640 (Gibco, Germany) supplemented with 10% (v/v) FBS, L-glutamine (2 mM), penicillin G (100U/mL) and streptomycin (100 µg/mL). The cells were cultured at 37 °C in a humidified incubator with 5% CO_2_. 

*Cytotoxicity assay*

*In-vitro* cytotoxicity of the peptide derivative on cells was assayed using the MTT [3-(4, 5-dimethylthiazoyl-2-yl) 2, 5 diphenyltetrazolium bromide] method (25). HT-29 cells were plated in a sterile 96 wells polystyrene microplate (10^4^- 10^5^ cells/well). Microplate was incubated at 37 °C in 5% CO_2 _atmosphere for 48 h. After removing the medium, each well loaded with 100 μL of fresh medium and 20 µL of different concentrations of the peptide in sterile ultra-pure deionized water in range from 0.1 to 250 µM. The wells without peptide were used as a negative control. Following 24 h incubation under the above mentioned conditions, the medium removed by vacuum and then filter-sterilized MTT (100 μL, 5 mg/mL DMSO) was added to each well and incubated for 3 h. The media was removed and the content of the wells for dissolving formazan crystal was mixed with 100 μL DMSO. Optical density was measured at 540 nm by using microplate reader and cell viability percentage was calculated by the absorbance ratio of cells treated with peptide to untreated cells dissolved in complete media. The experiment was performed in triplicate format for each peptide concentration under the same conditions.

*Hemolysis assay*

1 mL of fresh human blood from a healthy donor (age 48, male) with sodium citrate as anticoagulant was used for measuring the peptide derivative hemolytic activity. The prepared blood was centrifuged at 500 g for 5 min at 5 °C for the separation of red blood cells (erythrocytes). The separated red blood cells were washed three times with sodium phosphate saline solution (PBS, pH 7.3) and then the blood sample was diluted to a final concentration 5% (v/v) with PBS. Red blood cells suspension (100 µL) was added to 10 µL of different concentrations of the peptide derivative ranging from 0.1- 250 µM in centrifuge tubes and incubated at 37 °C for 1 h. Each peptide concentration was tested in triplicate format on microplate. 1%Triton X-100 solution and PBS solution were used as positive and negative control respectively. After incubation, the tubes were centrifuged at 1000 g for 5 min. 100 µL of the supernatant were added to the wells of 96- well microplate. The absorbance of the samples was measured at 540 nm by using microplate reader. The percentage of hemolysis was calculated by following formula as described by Hemmami *et al.* (26). Hemolysis (%) = [(mean Absorbance value of treated sample × mean Absorbance value of negative control) / mean Absorbance value of positive control)] × 100.

*Statistical analysis*

Statistical evaluation was carried out by using the SPSS 11.5 version for windows. All results were expressed as mean values ± SD (n = 3) for three independent experiments. Statistical significance was defined as *p* < 0.05.

## Results

*Peptide designing and synthesis*

**Figure 1:**
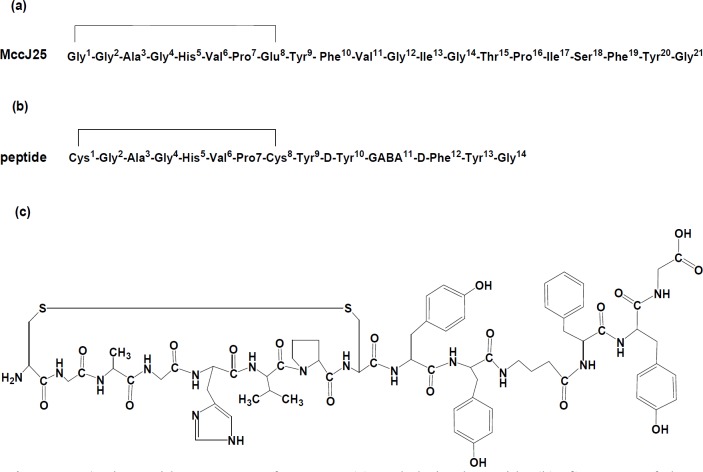
Amino acid sequences of MccJ25 (a) and derived peptide (b). Structure of the synthetic derived peptide (c)

In this study, a new cyclic peptide derivative comprising 14 amino acid residues based on the primary structure of MccJ25 lasso antimicrobial peptide with some modification was chemically synthesized according to solid phase peptide synthesis (SPPS) strategy. The structure of the synthesized peptide was as follows: [*cyclo *(Cys^1^-Gly^2^-Ala^3^-Gly^4^-His^5^-Val^6^-Pro^7^-Cys^8^)-Tyr^9^-D-Tyr^10^-GABA^11^-D-Phe^12^-Tyr^13^-Gly^14^] (Figure1). This peptide was designed by breaking the peptide bond between Phe^10^ and Val^11^ and deletion eight amino acid residues from Val^11^ to Ser^18^ of native MccJ25 sequence and insertion of gamma amino butyric acid (GABA) as a three-carbon chain spacer in backbone of the peptide. Furthermore, the target peptide was obtained by substituting of two D-form amino acids instead of L-form, i.e; D-Tyr^10^ (instead of Phe^10^) and D-Phe^12^ (instead of Phe^19^) in peptide structure. On the basis of the strict lariat protoknot structure of MccJ25 for maintaining the core section and its role as antimicrobial peptide, the lariat ring was synthesized in cyclized form. An intera-peptide disulfide bond was introduced between Cys^1^ and Cys^8^ which were substituted instead of Gly^1^ and Glu^8^ in the native MccJ25 sequence.

*Qualitative HPLC analysis*

**Figure 2:**
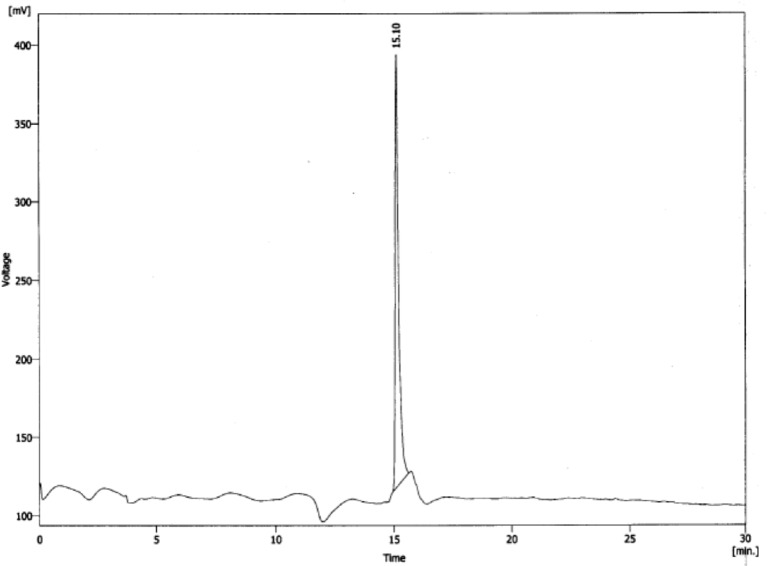
Reverse phase HPLC chromatogram of purified MccJ25-peptide derivative

For purification of crude peptide mixture, a semi-preparative RP-HPLC using column C_18_ with a mobile phase of 0.1% trifluroacetic acid (TFA)/ H_2_O as solvent A and acetonitrile as solvent B was employed. After multi run purification, analytical HPLC system showed the presence of a sharp peak (> 99.8% purity) as seen in Figure 2. The results revealed that the preparation of the peptide by solid phase method on trityl resin was successful by supplying an overall yield more than 60%, based on the removal of the Fmoc groups at first. The sharp peak, defined MccJ25-peptide derivative with retention time 15.10 min is the desired peptide component, representing the high yield in the chromatogram as determined by the percent peak area.

*Characterization of MccJ25-peptide derivative*

**Figure 3:**
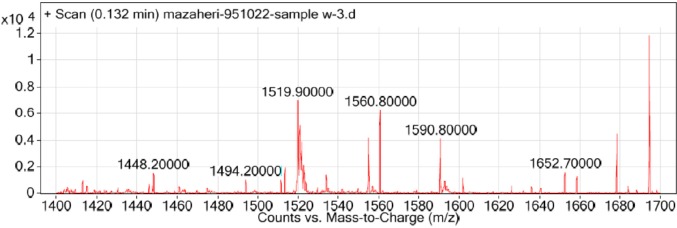
Mass spectrum analysis of the prepared peptide derived from MccJ25 in positive electrospray ionization (ESI) mode. Peaks at m/z 1519.9 and 1560.8 correspond to molecular ions [M+H] + and [M+CH3CN+H] + respectively

**Figure 4:**
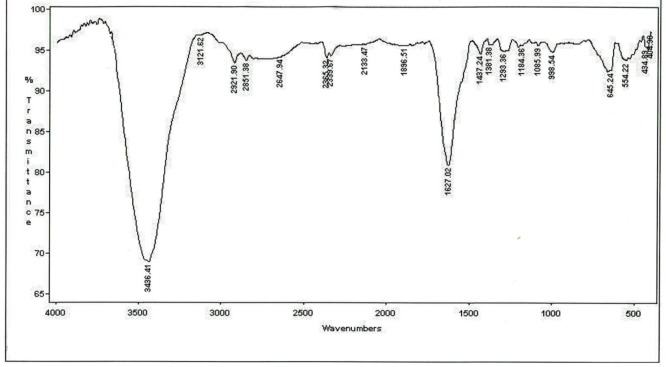
Infrared spectrum of the prepared peptide derived from MccJ25. (the details for IR spectrum characterization have been described in the text)

The LC-mass spectrometry for characterization of the correct mass of peptide was performed in positive ESI mode (Figure 3). The ESI mass spectrum from 1500 to 1600 mass to charge ratio (m/z) showed all the ionized molecules present in the peptide sample. Calculated mass for this new peptide derivative is 1518.6 gmol^-1^ and LC-MS analysis confirmed a [M+H] + molecular ion of 1519.9 (m/z). The FTIR analysis for structural characterization of peptide derivative is shown in Figure 4. The amide I, II, and III bands are the most used IR bands to reveal the structural properties of the proteins and peptides. The significant absorption band of amide I at 1627.02 cm^-1^ is attributed to C = O stretching vibration, indicating the presence of β-sheet structure. The observation of this band in the region 1600-1690 cm^-1^ is consistent with both theoretical calculations and experimental studies on a large number of peptides and proteins. The typical signals at 1437.24 cm^-1^ and 1293.36 cm^-1^ are assigned to the amide II and amide III bands respectively, due to C-N stretching vibration in combination with N-H bending. The absorption peaks at 2851.38, 2921.90, and 3121.62 cm^-1^ are characterizing the spectra of the C–H stretching band of the –CH2 and –CH3 stretching vibrations of the alkyl groups. The C–N absorptions are observed in the range of 1200 to 1350 cm^-1^ (aromatic) and 1000 to 1250 cm^-1^ (aliphatic). The wide and strong band at 3436.41 cm^-1^ originates from the O-H stretching vibration. The absorption band at 645.24 cm^-1^ was assigned to the C-S stretching vibration. The observed peak at 554.22 cm^-1^ was corresponded to the presence of disulfide bond (S–S).

*Biological activity of MccJ25-peptide derivative*

*Determination of MIC and evaluation of antimicrobial activity*

**Figure 5:**
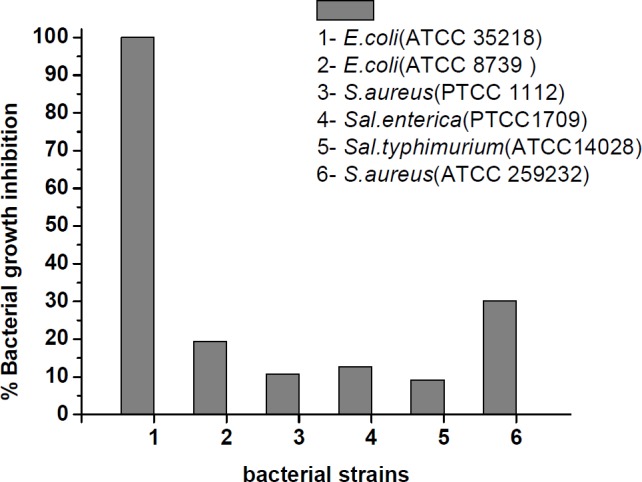
Inhibition of bacterial growth by the synthesized peptide at concentration of 250 µM after 24 h incubation at 35-37 ˚C

MICs were used for measuring the antibacterial efficacy of the peptide derivative against bacterial strains. MIC was considered as the minimum concentration of peptide that caused 100% growth inhibition and subsequently, there was no increase in the turbidity of each well when measured at 630 nm (was OD_630 _of 0.08-0.1). The MIC values are presented in Table 1. As shown in Table 1, the most sensitive bacterial strain is *E. coli* (ATCC 35218).The MIC value for this strain was determined of 3.9 µM while the MIC values for the other bacterial strains were values > 250 µM. In addition, it was remarkable that gentamicin showed the same activity against *E. coli* (ATCC 35218). The bacterial growth inhibition percentage in presence of the peptide at the highest concentration (250 µM) is illustrated in Figure 5. With the exception of *E. coli* (ATCC 35218), the highest and the lowest bacterial growth inhibition at concentration 250 µM of peptide were 30.47% and 9.73% that belong to *Staphylococcus aureus* ATCC 259232 and *Salmonella typhimurium,* respectively after 24 h incubation at 35-37 ˚C.

*Cytotoxicity results*

**Figure 6:**
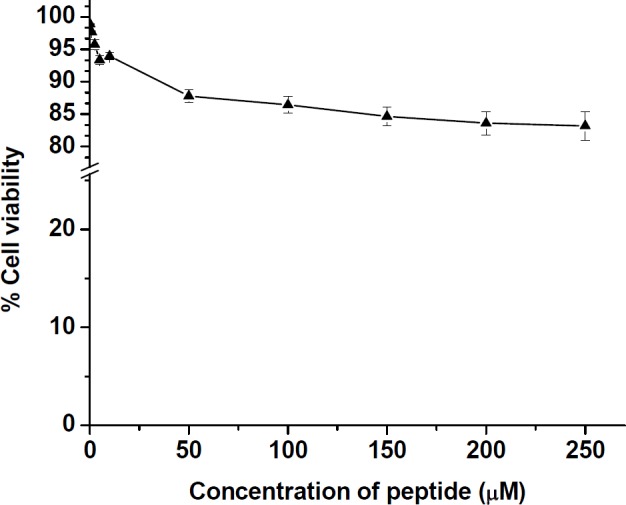
Cell viability (%) of HT-29 cell line after incubation with different concentrations of the synthesized peptide for 24 h incubation at 37 °C in 5% CO_2_ atmosphere. The values were calculated as mean ± SD of triplicate independent experiments

The cellular toxicity of the peptide derivative toward HT-29 was determined by MTT test. As shown in Figure 6, at various concentrations ranging from 0.1 to 10 µM of the peptide, the cell viability was estimated to remain more than 93 ± 0.5% with passing time for 24 h. Even at high concentration of peptide up to 200 µM, cell viability was still as high as 83.28 ± 0.3%, suggesting the toxicity of the peptide on the mammalian cell line is minimal at the tested concentrations ranging from 0.1 to 200 µM of peptide.

**Figure 7:**
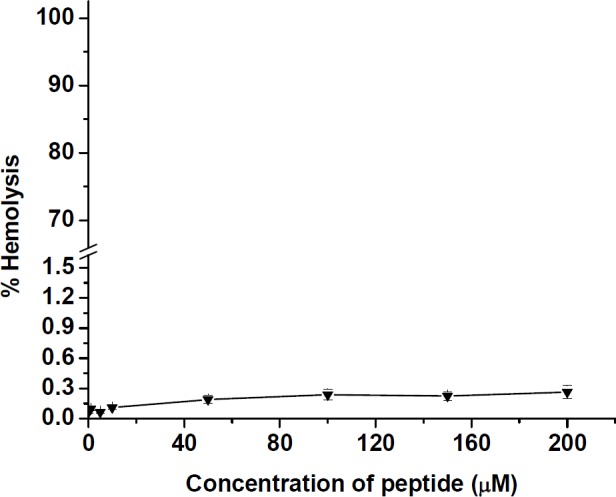
Hemolysis of human erythrocytes by different concentrations of the synthesized peptide. The values were calculated as mean ± SD of triplicate independent experiments

**Table 1:** Minimum inhibitory concentration (MIC) of the synthesized peptide against some bacterial strains

**Bacterial strain**	**MIC peptide (µM)**	**MIC Gentamicin (µM)**
*Escherichia coli *ATCC 35218	3.9	3.9
*Escherichia coli *ATCC 8739	>250	500
*Salmonella enterica *PTCC 1709	>250	250
*Salmonella typhimurium *ATCC 14028	>250	_
*Staphylococcus aureus *ATCC 25923	>250	500
*Staphylococcus aureus *PTCC 1112	>250	_

Doubling dilutions of peptide were applied in broth microplate test. MIC was considered as the minimum concentration of peptide that caused 100% growth inhibition.

*Hemolysis results*

The hemolytic activity was assayed as a function of hemoglobin release by measuring the absorbance of 100 µL sample supernatant at 540 nm. The results of hemolysis test in Figure 7 showed that peptide had a very low activity toward human erythrocytes. At various concentrations from 0.1 to 200 µM of the peptide, a hemolytic effect was obtained at a level below 0.3% as reached to 0.26 ± 0.01% for 200 µM of peptide.

## Discussion

With foundation a synthetic peptide based on the structure of existing natural antimicrobial peptides, it is possible to explore derivatives of that peptide with different characteristics of antimicrobial, hemolytic, and toxicity activities. In addition to these, it also provides an insight into the mechanism of action of the peptide. However, so far, many peptides with high potential activity have been made based on natural peptides to improve the biological factors such as stability and specificity of the peptides. 

Our study provided an opportunity to detect new synthetic peptide derived from a natural antimicrobial compound. In this study the antibacterial activity of a shortened sequence has been evaluated since cleaved MccJ25 has been shown previously to be antibacterial. Structure activity relationship (SAR) studies of MccJ25 have suggested that the lactam ring region (amino acids 1-8) plays distinct roles in the peptide antimicrobial activity and the C-terminal glycine was found to be important for RNAP inhibition (32, 33). Moreover, pervious results indicated that residues 11-15 located within the loop segment of the MccJ25 tail can be deleted, substituted, or chemically modified without loss of function (32, 34-38). Based on the finding mentioned above, for the synthesized peptide N-terminal and C-terminal sequences were maintained intact and GABA (gamma amino butyric acid) as a spacer was inserted instead of residues 11-18. Furthermore, peptide must be altered to be resistant against proteases or peptidases in serum or tissues. Chymotrypsin and elastase which are proteases synthesized by pancreatic acinar cells and secreted in the small intestine, are responsible for cleaving peptide bonds in hydrophobic amino acids such as phenylalanine and tyrosine (39, 40). To overcome the proteolytic cleavage of peptide and to increase the biological stability against proteases and proteolysis conditions two D-form amino acids (D-Tyr^10^ and D-Phe^12^) were inserted in the sequence. To our knowledge, this is the first report of preparation a new cyclic peptide derivative from antimicrobial MccJ25 lacking lasso structure and containing two D-amino acids residues and GABA (gamma amino butyric acid) spacer that retain its antibacterial activity. The peptide composed of 14 amino acids with different length and hydrophobicity from the native MccJ25. 

To perform a folded conformation in the prepared peptide structure, a disulfide bond was formed on linear peptide when it was cleaved from 2-chlorotrityl chloride resin and the de-protection of side chains was achieved during the peptide synthesis. The purification of crude peptide was successfully performed using semi-preparative RP-HPLC prior to structural characterization by LC-Mass and FTIR spectroscopy. Molecular weight determination by electrospray ionization mass spectrometry (ESI-MS) confirmed the correct preparation of peptide using Fmoc solid phase synthesis strategy. In addition, FTIR analysis revealed the significant absorption bands of amide I, II, III, and more detailed structural properties of peptide in reaction environment. 

Antimicrobial activity was analyzed based on the results of MIC by micro dilution assay. When analyzing MIC, it was revealed that among six bacterial strains, the peptide derivative exhibited effective activity against only *E. coli* ATCC35218 at range concentrations tested. Up to now, some different peptide analogues with new characteristic have been found for MccJ25. Soudy *et al*. (2012) synthesized six peptides derived from MccJ25 named 1-6 of different lengths and hydrophobicity. Among them two peptides 1 and 6 displayed good activity against *Salmonella newport*. Furthermore, Hammami *et al*. (2015) chemically synthesized a number of analogue peptides with different lengths without the lasso structure. Based on the results obtained by the authors, three various peptides derived from MccJ25 had inhibitory effects on *Salmonella enterica* and *E. coli* but all the three peptides were less potent than native MccJ25. However, the growth inhibition of all the tested bacterial strains documented at Figure 5 suggesting that the prepared peptide was able to interact with the bacterial cellular membrane to inhibit the growth due to its structural physicochemical properties. The peptide had a low inhibitory effect against *Salmonella* species whereas had high potency against *E. coli *ATCC35218 (MIC = 3.9 µM). Interestingly, it was observed that the peptide was about 3 fold more potent against *S. aureus* (ATCC 259232) compared to *Salmonella *species (*Sal.enterica* with value of 12.22% and *Sal.typhimurium* with value of 9.73% inhibition growth). 

This finding is different from the results, previously reported that MccJ25 is active against *Enterobacteriaceae *family, including *Escherichia coli* and *Salmonella* strains (11,14), While Vincent *et al.* also demonstrated that some strains of *Salmonella typhimurium*, *Salmonella derby*, and *Salmonella enteritis* were completely resistant to MccJ25 (11). However, it is important to note that in comparison with native MccJ25, the prepared peptide with a disulfide bond acts selectively on the tested bacterial strains and also confirmed that lasso folding in MccJ25 structure may not be required for antimicrobial activity of the peptide. In cell toxicity assay, this is obvious that one of the most important limitations for development of AMPs in clinical application is the toxicity to human cells (41). The recent studies have shown that MccJ25 has no cytotoxicity due to the lack of access to mammalian cell membranes. Our results confirmed the previous findings related to the cytotoxic effect of MccJ25 (25, 42). It was observed that the prepared peptide displayed very low toxicity (more than 83% cell viability) to mammalian cells HT-29 up to the highest concentration tested (200 µM) compared with > 80% cell viability at low concentration (100 µM) obtained previously for native MccJ25 and the synthetic peptide derivatives in MCF-7 and MDA-MB-435 cancer cell lines. However, the low cytotoxicity of the prepared peptide as an anticancer agent could be related to inability to permeate human cancerous cells (41). In comparison to other different peptides derived from MccJ25 that were synthesized by Hemmami *et al*. (26), the hemolytic activity of our peptide toward human erythrocytes was very insignificant. The results of toxicity against human erythrocytes demonstrated that the prepared peptide derivative did not display cytotoxic effects at the tested concentrations from 0.1 to 200 µM of peptide on these cells. On the other hand, the peptide has no effect on human erythrocytes, therefore, it is safe on human red cells.

## Conclusion

In this study, a modified peptide derivative was designed from one of the most known microcins from *Entrobactericaea *family, microcinJ25. However, the present MccJ25-derived peptide showed poor activity against some tested bacterial strains, but it was highly active against *E. coli* ATCC 35218. For this new modified peptide, cancerous cell (HT-29) toxicity was not observed. Based on the obtained results, this compound could be considered for further investigation as a new antimicrobial agent in treatment of infectious diseases. On the following research, more studies including mechanism of action of the designed peptide on microorganisms and the other applications are being planned for implementation in near future.
